# Sleep complaints and daytime sleepiness among pharmaceutical students in Tripoli

**DOI:** 10.3402/ljm.v7i0.18930

**Published:** 2012-10-30

**Authors:** Yousef A. Taher, Awatef M. Samud, Aya H. Ratimy, Areeje M. Seabe

**Affiliations:** 1 Department of Pharmacology and Clinical Pharmacy, Faculty of Pharmacy, Tripoli University, Tripoli, Libya; 2 Department of Anaesthesia and Intensive Care, Faculty of Medical Technology, Tripoli University, Tripoli, Libya

**Keywords:** college students, sleep habits, sleep disorders, Epworth Sleepiness Scale

## Abstract

**Background:**

The effect of sleep difficulties has achieved a great deal of attention recently, with university students considered as a homogenized population, particularly affected by sleep habits.

**Aim:**

The objective of this study was to investigate whether Libyan college students experience sleep disturbance during their academic programmes.

**Methods:**

A cross-sectional survey was conducted in the college of Pharmacy, Tripoli University, during February 2010. A total of 201 students, including 179 females (89.05%) and 22 males (10.95%), were recruited from different academic levels. Data were collected using a structured questionnaire and included a number of life-style variables. Epworth Sleepiness Scale (ESS) was used for the assessment of daytime sleepiness.

**Results:**

This study showed that the total sleep time (TST) on a weeknight was 6.40 h and 67 students reported napping during daytime. The TST plus naps totalled 7.39 h. Out of eight possible dozing situations, we found that the mean score for ESS was 8.78. In addition, 79 students showed an ESS score of >10. Furthermore, our results showed that the majority of students (>92%) reported poor sleep satisfaction with quality and duration of sleep hours. Thinking about difficulty of study but not increasing education programs or tea/coffee consumption is associated with sleep difficulties reported. Moreover, 77.6% of students reported an irregular sleep–wake schedule.

**Conclusion:**

These findings indicate that students experienced excessive daytime sleepiness. The TST of pharmaceutical students in Libya, as in other developing countries, is less than those reported by Western students. Students experienced various environmental demands during their college years and, their quality of sleep was negatively affected.

Sleep is considered a natural periodic state of rest for the brain, mind, and body. In normal day–night conditions, the sleep–wake sequence is 24 h long. Consequently, bodily systems, including the immune, digestive, and cardiovascular systems are believed to be heavily influenced by sleep habits (1, 2). Furthermore, Riemann and Voderholzer have shown that sufficient sleep is correlated with both normal physical and mental functions, thus improving learning skills (3). However, in addition to the actual amount of sleep, each person also needs a proper sleep pattern, and wakefulness varies widely between individuals and is influenced by many factors. It has been shown that exposure to physical and psychiatric disorders, environmental stress, as well as shift work can result in partial loss of sleep (4). A number of medical problems, including high blood pressure, heart diseases (5, 6), obesity, and diabetes (7, 8) have been shown to be associated with sleep deprivation. Actually, a lower sleep quality, recognized as a sleep disorder, induces impaired functioning and excessive sleepiness (1). In general, a person needs more sleep if he or she has not had ample sleep on the previous night. A lack of sleep creates a ‘sleep debt’, which is much like being overdrawn at a bank (9). Although people can get used to a sleep-deprived schedule, it will, however, result in their judgement, reaction time, and other functions becoming impaired (10).

University students are considered as a young healthy population with few sleep problems (11). However, owing to the transitional nature of college life, they face different physical and psychological pressures. Students need a great deal of knowledge and extensive training to obtain their qualification. Hence, students are often challenged with remarkable loads. In a limited period of time, students try to acquire adequate scientific knowledge to maintain a high level of academic achievement, whilst adapting to an ever-changing environment and learning new skills. Accordingly, there is no doubt that having enough sleep improves one's mood and makes one feel less stressed (12), whereas a chronic lack of sleep increases the risk for accidents, injuries, and illnesses. Unfortunately, college students do not recognize that bad sleep habits might play a role in their academic difficulties. In fact, studies have shown that students who have normal night sleep performed considerably better than those sleep-deprived students (13, 14). Therefore, the question was how much sleep does a student need? Overall, sleep-related problems among college students have been evaluated by a number of studies (15–17). In addition, there is little information regarding sleep disturbance and sleep behavior of university students (16, 17). Also, in Libya, the sleep patterns and factors contributing to sleep disorders of university students have not been well studied. Hence, the main scope of the current study was to characterize the sleep patterns and describe the prevalence of daytime sleepiness and the associated factors among a relatively homogeneous population of pharmaceutical students, at different academic levels, by a questionnaire specifically prepared for the study of sleep characteristics.

## Students, materials and methods

### Data collection

A cross-sectional descriptive study among resident Libyan undergraduate students at the faculty of Pharmacy, Tripoli University, Tripoli, Libya, was conducted over a 4-week period during February 2010. The participants included in this study were healthy pharmaceutical students aged between 20 and 24 years, registered as full-time students during the study. Similar to other studies (16), students who reported that they were repeaters, complaining of a chronic disease, often making night calls or are not considered as full academic subjects were excluded from the study. In addition, none of the participants drank alcohol. The students were randomly recruited, by meeting them at the library, at different times of the day and several times a week, through face-to-face interviews. Trained female students (the co-authors) contacted participants and illustrated to them the aim of the study and the protocols to assure completeness and accuracy of data collection. The survey included a sample of 179 female (89.05%) and 22 male (10.95%) students, which accords for the gender distribution at the college (84.35% females, 15.64% males), at different academic levels [first (L1), second (L2), third (L3), and fourth (L4)]. These 201 students were considered to be representative of the students in this college. During this initial interview, students were asked to explain their sleep habits and other factors on weekdays, as illustrated in the questionnaire. In addition, the most common sleep disorders and medical problems were recorded. First academic lectures for the day began between 07:30 and 08:00 a.m. Informed consent from each participant, as well as local university ethical approvals were obtained.

### Survey form and the questionnaire

The survey was performed by a face-to-face interview technique using a well-prepared questionnaire, translated into simple Arabic to ensure its comprehensibility. The students returned completed questionnaires, including information regarding health behavior. The questionnaire consisted of two sections, which were taken from other published surveys so that, in addition to fulfilling our study objectives, a comparison could be made with the results from other countries. The first section touches upon age, level of education, naps, total sleep time (TST) at night per week, and potential factors that might influence daytime sleepiness, with answer options being true and false, quantifying the participants’ sleep on duration and quality. The second section of the questionnaire, for the measurement of general level of daytime sleepiness, consisted of items divided logically using an international, reliable, validated, and specialized questionnaire, the Epworth Sleepiness Scale (ESS), proposed previously by Johns (18). The ESS covers eight different situations of everyday life activities. Students were asked to answer the overall ESS components and indicate whether they had come across each complaint or difficulty within the previous month. The ESS score (range 0–24) is an indication of the tendency to fall asleep in those situations. The studies by Johns estimated total ESS score >10 as increased daytime sleepiness (18). For each question, the manifestation of the experienced problem by a student was estimated as an important score and the mean extents were calculated. ESS questions were completed and statistically evaluated for comparison. The TST and nap duration was calculated according to the information extracted from each participant's feedback.

## Data analysis

Values are presented as mean±SE unless otherwise stated. The results were analysed statistically to obtain the sociodemographic characteristics of the subjects. Chi-square and Fisher's exact test were used to test for important differences between groups, as appropriate. Mann–Whitney or Student *t*-test was used to test for significant difference between groups, as applicable. The Kruskal–Wallis one-way analysis of variance (ANOVA) was used to compare groups. The strength of concordance between the variable rationales was evaluated using Spearman's rank correlation coefficient. Overall, *P*-value of <0.05 was considered significant. To evaluate the strength of association between the independent and the dependent variables, the logistic regression type of analysis was used. Odds ratios (OR) and 95% confidence intervals (CI) were calculated to quantify these associations. Analyses of data were performed using GraphPad Prism (GraphPad Software Inc., version 3.0, San Diego, USA).

## Results

Of the 302 questionnaires distributed, 273 students (90.40%) agreed to participate and responded to the study. Of those, 201 questionnaires (73.63%) were properly completed by full academic students who met the overall inclusion criteria. Distribution of academic levels was as follows; L1: 38.31%; L2: 23.38%; L3: 18.41%, and L4: 19.90% (Table 1). The mean age (±SD) of the students was 20.77±1.84 years. Of the researched group, the present study showed that the TST was 6.40±0.10 h a night/week (Table 1), suggesting insufficient sleep (9). In addition, 33% of our sample of college students affirmed napping many times per week. The mean duration of daytime sleep (nap) in the whole group was 2.34±0.12 h per week. A one-way analysis of variance revealed that L1 students experienced duration of a nap significantly more than L2 and L3 students. Furthermore, our findings showed that the TST + naps were 7.39±0.14 h (Table 1). However, no important variations were observed among the four studies grouped in TST and TST + nap (Table 1). Similar findings were noticed in TST, duration of nap, and TST + nap, when L2 pharmaceutical students were compared to L2 medical, L2 dental, or L2 techno-medical students (data not shown).

**Table 1:** Sleep characteristic of the students

Characteristics	L1 (*n*=77) 38.31%	L2 (*n =* 47) 23.38%	L3 (*n*=37) 18.41%	L4 (*n*=40) 19.90%	Overall (*n*=201)
Mean age±SD (years)	19±0.77	20±1.30	21±1.12	22±1.60	20.77±1.84
TST (h/night/week)	6.27±0.19	6.52±0.22	6.24±0.15	7.10±0.24	6.40±0.10
Students who napped during the daytime (%)	(17) 25.37%	(15) 22.39%	(15) 22.39%	(20) 29.85%	(67) 33.33%
Duration of nap (h)	2.57±0.27*	2.10±0.26	2.04±0.18	2.23±0.20	2.34±0.12
TST plus nap (h)	7.16±0.25	7.37±0.27	7.33±0.20	7.18±0.25	7.39±0.14
ESS score	8.40±0.35	8.42±0.49	9.84±0.55	8.97±0.53	8.78±0.23
EDS (ESS > 10)	(28) 35.44%	(16) 20.25%	(19) 24.05%	(16) 20.25%	(79) 39.30%
Students who wake up late	(9) 22.5%	(11) 27.5%	(6) 15.0%	(14) 35.0%	(40) 52.63%
Students who reported poor sleep satisfaction	(70) 37.63%	(47) 25.27%	(35) 18.82%	(34) 18.28%	(186) 92.54%

TST: total sleep time; ESS: Epworth Sleepiness Score; L1, L2, L3, and L4: first, second, third, and fourth academic levels; EDS: excessive daytime sleepiness.

* *P <* 0.05 compared to L2 and L3.

The present study showed that about 44% (88 students) reported praying Fajr prayer on time and only 22.4% (45 students) had a regular sleep–wake schedule (data not shown). In addition, the majority of students (77%) reported that although they went to bed later on weekends than weekdays, they also woke-up next morning much later than on weekdays (data not shown). All students denied having sleep-inducing medicine or alcohol.

The mean ESS total score for the students was 8.78±0.23 (Table 1), indicating occasional sleep difficulties. Statistically, no significant difference in ESS score was noticed between all individual groups. Results of the ESS showed that when students were sitting for reading, lying down for a rest, or sitting quietly after lunch, they experienced moderate to high chance of dozing than when they were sitting inside a car, which had stopped briefly in traffic, or when sitting for chatting with someone (*P*<0.001, Table 2). In addition, close to 50% of the students reported a slight to moderate chance of dozing when they were sitting inactive in a public gathering (Table 2). In contrast, 1-h of travelling, without a break, by car or watching television resulted in fewer chances of dozing (29 and 34%, respectively, Table 2). Overall, based on an ESS score of >10, the current study showed that 79 students experienced excessive daytime sleepiness (EDS) (Table 1). The TST reported by students who had EDS was comparable to those who did not have EDS (6.21±0.17 h vs. 6.50±0.13 h, respectively, *P*=0.17, data not shown). Nonetheless, while the students used naps as a compensatory occasion for EDS and low TST, we also found that there are no significant differences in TST plus nap compared to those without EDS (data not shown).

**Table 2:** Students’ responses to items in the Epworth Sleepiness Scale

	Score	Interquartile range		
		
Sleepiness scale	‘who would never doze’ (*n*) mean (95% CI)	25th centile	Median	75th centile
1. Sitting and reading	(8) 1.7 (1.6–1.8)***	1	2	2
2. Watching T.V.	(69) 1.0 (0. 9–1.1)	0	1	2
3. Sitting inactive in a public gathering	(100) 0.8 (0.6–0.9)	0	1	1
4. As a passenger in a car for an hour without a break	(59) 1.3 (1.2–1.5)	0	1	2
5. Lying down for a rest in the afternoon when circumstances permit	(25) 1.7 (1.6–1.9)***	1	2	3
6. Sitting and talking with someone	(155) 0.3 (0.2–0.4)	0	0	0
7. Sitting quietly after lunch without alcohol	(38) 1.6 (1.5–1.8)***	1	2	2.5
8. In a car stopped briefly in traffic	(158) 0.3 (0.2–0.4)	0	0	0

Range 0–3: (0) would never doze; (1) slight chance of dozing; (2) moderate chance of dozing; (3) high chance of dozing. CI: confidence interval

*** *P <* 0.001 compared to items number 6 and 8, Kruskal–Wallis test.

Out of the 201 respondents who answered the questionnaire, 112 students had sleep disturbance (Table 3). This is manifested in rated scores in response, the response being yes/no, to a question regarding the difficulty in sleep, subjective sleep quality, and sleep duration. The data showed that level of education was not a significant factor for insufficient sleep (*χ* ^2^=0.54, Table 3). The prevalence of sleep disturbance was highest among L1 students, whereas L3 students were less prone to have sleep disturbance with the lowest 95% CI [OR = 1.02 (0.48–2.20)] compared to L1 students (Table 3). Thirty-eight percent of our sample of college students (76 students) reported tardily to school several times a week (data not shown). As shown in table 1, 40 students attributed these missing lectures to waking up late. In spite of this, there is no significant variation in TST among subjects who were coming on time and those who were coming late (6.55±0.13 vs. 6.21±0.18 h, respectively).

**Table 3:** The association between sleep disturbance and academic levels among respondents

Academic level	Sleep disturbance (*n*=112) n (%)	No sleep disturbance (*n*=89) n (%)	Odds ratio (95% CI)	*P* *
First year (L1)	40 (35.71)	37 (41.57)	1.69 (0.80–3.54)	0.54
Second year (L2)	31 (27.68)	17 (19.10)	1.16 (0.52–2.56)	
Third year (L3)	20 (17.86)	16 (17.80)	1.02 (0.48–2.20)	
Fourth year (L4)	21 (18.75)	19 (21.35)		

* *χ* ^2^ test.

More than 92% of the participants (Table 1) reported poor sleep satisfaction with regard to quality (64.68%) and quantity (87.07%) of sleep. The most dissatisfied students were L1 at 37.63%, followed by L2 students at 25.27%. The least reported sleep dissatisfaction was among L4 students at 18.28% (Table 1). As shown in table , students who were sleep-dissatisfied attributed this to the following different rationales: (1) thinking about the difficulty of their study (82.09%); (2) poor personal organization (85.07%); (3) studying at the expense of sleep (56.72%); and (4) drinking stimulants such as tea and coffee (66.67%). Logistic regression showed that students who reported thinking about the difficulty of study were approximately tenfold more likely to develop sleep dissatisfaction (OR = 9.70, 95% CI: 3.06–30.73; *P <* 0.001) compared to those students who did not have any trouble with their study (Table 4). For poor personal organization, OR was 4.50 (*P <* 0.05), indicating that since there was irregular sleep schedule, the odds of showing sleep dissatisfaction increased almost fivefold. While students who drank tea or coffee or who studied at the expense of sleep were two- to threefold more prone to experience pitiable sleep satisfaction [(OR = 1.84, 95% CI = 0.64–5.31); (OR = 2.83, 95% CI = 0.93–8.61); respectively] as compared to other unit groups. However, no statistical significant difference for sleep disturbance was found between those with excessive and poor tea/coffee consumptions (*P =* 0.66, data not shown). Furthermore, the present study showed that consumption of tea/coffee negatively correlated with all the outcome rationales (not significant, Table 5). In contrast, a good positive inter-correlation was found between another three rationales, using Spearman's rank correlation coefficients (*P <* 0.001, Table 5).

**Table 4:** The association between sleep dissatisfaction and predictors of sleep dissatisfaction among respondents

		Odds ratio (95% CI)	
			
Rationales	% prevalence of reason (*n*)	Sleep dissatisfaction	*P* *
Thinking about difficulty of study	82.09 (165)	9.70 (3.06–30.73)	0.0001***
Poor personal organization	85.07 (171)	4.50 (1.47–13.77)	0.013*
Studying at the expense of sleep	56.72 (114)	2.83 (0.93–8.61)	ns
Tea or coffee intake	66.67 (134)	1.84 (0.64–5.31)	ns

Calculation based on presence of at least one reason/totally absence of reasons

* Fisher's exact test. ns = not significant

* *P <* 0.05

*** *P <* 0.001.

**Table 5:** Spearman rank correlation matrix (two-tailed) calculation based on presence/absence of rationales

Rationales		(1)	(2)	(3)	(4)
Thinking about difficulty of study	(1)	1.00			
Poor personal organization	(2)	0.24*	1.00		
Studying at the expense of sleep	(3)	0.30*	0.11 ns	1.00	
Tea or coffee intake	(4)	−0.02 ns	0.01 ns	0.06 ns	1.00

* *P <* 0.001 compared to rationale (1). ns = not significant.

## Discussion

Sleep complaints are common conditions that can be overcome. However, worldwide, sleep habit investigators are debating as to how much sleep per night a healthy creature should have. In addition, because of the steady rise in work needs, sleep insufficiency has become a global phenomenon (19). It has been shown that young adults need an average TST of 7.5 h on weeknights and a bit more, that is, 8.5 h during weekend nights (17). Accordingly, life constraints affect those students, who are dealing with curative sciences, making them experience irregular sleep habits compared to individual sleep needs.

Herein, responses to the survey showed that sleep deprivation, both in quality and duration, is much more common than was once thought, with 56% of the participants reporting sleep disturbances. Data in the current study showed that the TST is short compared with several other studies of young adults (17). Indeed, our data have shown that the average TST during weeknights was 6 h and 40 min, which is notably 1 h and 20 min less than that reported in studies from developed industrialized communities (17). Buboltz *et al*. (17) have shown a mean duration of sleep of 8 h and 2 min per night in 191 psychology students in the USA, which is considered normal for most individuals. The majority of participants in this study did not sleep by napping; however, sleep extension occurred on non-school nights for 77%. Increased sleep duration on the weekend may indicate their actual sleep demand and may also be a signal for ‘catch-up’ sleep to substitute for the lack of sleep on weekdays. Also, the reported napping during the day by some students might be considered as further compensatory actions. Consistent with the findings by Monk *et al*. (20), we did observe a decrease in the mean duration of night sleep in students who nap compared to the whole subjects. Concurrently, in the nappers, adding the nap sleep hours to the night sleep hours increased the TST by 99 min, and accordingly, the mean TST of all the subjects was enhanced by 59 min. In general, even during daytime napping, students slept considerably less than individuals in this age group (17), a pattern not unique to this sample. At the same time, our findings indicated that students are indifferent to their sleep problems since they reported that they were not seeking medical assistance.

The current study is in accordance with several studies that have evaluated sleep habits and patterns among university students. Such studies showed that Saudi healthcare workers slept for 6 h and 30 min on weeknights (21). Lowry *et al*. (14) have shown that the extent of sleep is strongly associated with academic performance and also with various undesirable effects that might be life threatening. Furthermore, lack of sleep can induce decreased cognitive performance, decreased thinking and concentration (10). The significance of these consequences is more critical if the subject is a university student. Thus, our findings corroborate the concern that many students experience sleep deprivation compared with internationally assumed sum of sleep hours. In addition, it indicates a crucial necessity for a nationwide study on sleep behaviors among ‘Libyan’ people.

In the present study, about 50% of students experienced sleep disturbance during the educate-nights, and these gathered the ESS principles for sleep difficulties. The percentage of students in the current study who scored a global sum of ESS of more than 10 points (considered to have EDS) were 39.30%, slightly higher than a representative sample from Saudi medical students who reported only 22.4% of 129 subjects (16). The present data indicated that sitting and reading increased daytime dozing to a great extent in students who had EDS. The exact nature of EDS in our students is difficult to interpret because of the presence of several confounding variables, such as poor personal organization, and approximately more than two-thirds of our subjects had irregular sleep schedules. In addition, although the prevalence of sleep disturbance was high among L1 students, we found that education programs and intervention are not the case here. This pattern is similar to that reported previously of a related study using an identical questionnaire (16).

It seems that, in Libya and other neighboring countries, sleeping lightly at midday is a cultural behavior. So, increases in the number of subjects who nap might indicate an increased bodily demand for more sleep owing to sleep deprivation (22). The current study revealed that 33% of students nap during the day. In Morocco, similar to the present study, a lower percentage (41%) of students, at the faculty of human sciences, reported daytime napping (23). Saudi medical students, in contrast, reported a high percentage (88%) of napping during the day (21). One possible explanation for this discrepancy is that Wali *et al*. (21) surveyed only male students whereas in our study, over 85% were females. Nonetheless, the possibility that gender factor might influence sleep habit is not clear since some studies (17), but not all (24), showed a significant difference between male and female subjects.

Epworth Sleepiness Scale is used for the assessment of daytime sleepiness among several age groups (18). Therefore, our findings can be considered as a traditional point estimate since the longitudinal information obtained is subjective and self-reported. Consistent with other studies (15, 21), our findings indicated that 79 out of 201 subjects scored an ESS of >10, an indicator of increased daytime sleepiness. Furthermore, we have also examined the actigraph records (ESS score) and noted that about half of the students misjudge the risk of dozing off, which may signify that the evaluated percentage of stated sleepiness is underestimated.

In addition to decreased sleep duration, the present study showed that participants also suffer from poor quality sleep. The prevalence of poor sleep satisfaction was comparable to values reported for Western peers (17). The experience of reduced sleep satisfaction possibly suggests some problems with students; those who accept shortage of sleep may act as if they are exhausted (17). There are numerous assumptions that sleep habits induce different behaviors, which eventually become habituated (25). Besides, although it cannot totally exclude that a subjective health had no impact on personal sleep need, however, for special reasons, the majority of students did not answer the question. The Fisher's exact test suggested no significant relationship with tea or coffee consumption, although the OR findings showed that students who take tea or coffee tend to report higher sleep complaints than those who did not. Therefore, it is not likely that tea or coffee intake is recommended as a sleep preventive measure on satisfaction. Unfortunately, however, not enough data are available on sleep disorders among the local communities to allow direct evaluation of potential factors that could contribute to this phenomenon. On the contrary, these data indicated that thinking about difficulty of their studies strongly induced poor sleep satisfaction and is therefore significantly associated with sleep difficulties. A positive association of thinking about difficulty of studies with sleep dissatisfaction was found in other studies (16). The present study, and others (16), revealed that students who reported higher sleep-self dissatisfaction tend to be less organized. Therefore, students’ sleep habits could be inversely related to environmental pressure and other difficulties during college years (16, 17).

In this study, several limitations have been counted during interpretation of our findings. First, since the study population was taken from the faculty of pharmacy, it does not represent all of the university students. Second, the self-reported questionnaire might contain inaccurate answers, although we tried to eliminate this defect by having direct contact with the participants. A third limitation is that we did not evaluate the sleep pattern on weekends. Fourth, since the present study is a cross-sectional study, the possibility of recall bias cannot be ruled out. Finally, the present study failed to investigate the effect of sleep difficulty on the student's academic achievement. However, our sample size matched the overall students for literacy rate. In addition, it demonstrated wide-range variation from several traditions and had a high response rate. Hence, the most noteworthy findings may represent crucial knowledge regarding students’ sleep patterns, and open the door for further research in this area.

In conclusion, the present study revealed that the TST among our college students is lower than that in Western countries but similar to other developing countries. Furthermore, it emphasized that: (1) Additional studies on a much larger scale are needed to provide more information. Such studies should be extended to look at factors that may influence sleep quality and students’ academic successes. (2) Helpful instructions, which may support students to minimize their sleep difficulties and prevent the negative effects of sleep deprivations on students’ academic performance, ought to be enlightened.
